# Linking Women Who Test HIV-Positive in Pregnancy-Related Services to HIV Care and Treatment Services in Kenya: A Mixed Methods Prospective Cohort Study

**DOI:** 10.1371/journal.pone.0089764

**Published:** 2014-03-19

**Authors:** Laura Ferguson, Alison D. Grant, James Lewis, Karina Kielmann, Deborah Watson-Jones, Sophie Vusha, John O. Ong’ech, David A. Ross

**Affiliations:** 1 Institute for Global Health, University of Southern California, Los Angeles, California, United States of America; 2 MRC Tropical Epidemiology Group, Department of Infectious Disease Epidemiology, London School of Hygiene and Tropical Medicine, London, United Kingdom; 3 University of Nairobi Institute for Tropical and Infectious Diseases, Nairobi, Kenya; 4 Clinical Research Department, London School of Hygiene & Tropical Medicine, London, United Kingdom; 5 Institute for International Health & Development, Queen Margaret University, Edinburgh, United Kingdom; 6 Mwanza Intervention Trials Unit, National Institute for Medical Research, Mwanza, Tanzania; 7 Department of Obstetrics and Gynaecology, University of Nairobi, Nairobi, Kenya; Johns Hopkins School of Public Health, United States of America

## Abstract

**Introduction:**

There has been insufficient attention to long-term care and treatment for pregnant women diagnosed with HIV.

**Objective and Methods:**

This prospective cohort study of 100 HIV-positive women recruited within pregnancy-related services in a district hospital in Kenya employed quantitative methods to assess attrition between women testing HIV-positive in pregnancy-related services and accessing long-term HIV care and treatment services. Qualitative methods were used to explore barriers and facilitators to navigating these services.

Structured questionnaires were administered to cohort participants at enrolment and 90+ days later. Participants’ medical records were monitored prospectively. Semi-structured qualitative interviews were carried out with a sub-set of 19 participants.

**Findings:**

Only 53/100 (53%) women registered at an HIV clinic within 90 days of HIV diagnosis, of whom 27/53 (51%) had a CD4 count result in their file. 11/27 (41%) women were eligible for immediate antiretroviral therapy (ART); only 6/11 (55%) started ART during study follow-up. In multivariable logistic regression analysis, factors associated with registration at the HIV clinic within 90 days of HIV diagnosis were: having cared for someone with HIV (aOR:3.67(95%CI:1.22, 11.09)), not having to pay for transport to the hospital (aOR:2.73(95%CI:1.09, 6.84)), and having received enough information to decide to have an HIV test (aOR:3.61(95%CI:0.83, 15.71)). Qualitative data revealed multiple factors underlying high patient drop-out related to women’s social support networks (e.g. partner’s attitude to HIV status), interactions with health workers (e.g. being given unclear/incorrect HIV-related information) and health services characteristics (e.g. restricted opening hours, long waiting times).

**Conclusion:**

HIV testing within pregnancy-related services is an important entry point to HIV care and treatment services, but few women successfully completed the steps needed for assessment of their treatment needs within three months of diagnosis. Programmatic recommendations include simplified pathways to care, better-tailored counselling, integration of ART into antenatal services, and facilitation of social support.

## Introduction

Global attention to HIV testing in pregnancy has focused almost exclusively on prevention of mother-to-child transmission (PMTCT) of HIV. [1] Attention is now increasingly being paid to linking women who are diagnosed with HIV in antenatal or delivery services (collectively “pregnancy-related services”) to long-term care and treatment for their own HIV infection. [2].

Based on the potential advantages to mother and infant of early initiation of maternal antiretroviral therapy (ART), [3] Kenya, along with many other countries, is considering the adoption of a policy of immediate lifelong ART for all women diagnosed with HIV during pregnancy (also known as “Option B+”). To maximize the potential benefits of Option B+, linkage into ART services following an HIV diagnosis in pregnancy-related services is required. However, a recent systematic literature review highlighted sub-optimal linkage into long-term HIV care and treatment services of women diagnosed with HIV during pregnancy. [4].

We carried out a retrospective cohort study in Naivasha Hospital, Kenya, between January 2008 and June 2010, finding high attrition along the pathway to HIV care and treatment: within six months of diagnosis with HIV in pregnancy-related services, only 153/892 (17%) women had registered at the on-site HIV clinic. Of 99 women with a recorded CD4 count, 53 were eligible for and only 21 (40%) initiated ART within six months of HIV diagnosis. [5] This review was based on manually-matched hospital records, potentially underestimating uptake of care in nearby health facilities.

We therefore recruited a cohort of HIV-positive women within pregnancy-related services in the same hospital and followed them prospectively, aiming to quantify uptake of long-term HIV care and treatment services while taking into account uptake of services at other nearby health facilities and to explore reasons underlying client attrition along the care continuum.

## Methods

### Ethics Statement

Ethical approval was provided by the University of Nairobi/Kenyatta National Hospital Ethics Review Committee and the London School of Hygiene & Tropical Medicine Ethics Committee. Information sheets were read to all participants and written informed consent was obtained at enrolment and again for qualitative interviews. This included consent for researchers to access participants’ medical records. Three women aged 16–17 participated, each of whom was deemed competent to provide informed consent; involvement of their parents was discussed and encouraged but refused by the participants. To preserve confidentiality, unique study numbers were assigned at enrolment and used in all active databases and documents. After the qualitative interviews, participants who were not engaged with HIV care were encouraged to attend clinic.

### Setting

This study was carried out in Naivasha District Hospital, Rift Valley Province, Kenya. Among women in Rift Valley Province, HIV prevalence is 6.3%, [6] while in the study hospital the estimated HIV prevalence of HIV among antenatal attendees is 4% (based on routine hospital data, 2009–10).

According to Kenyan national guidelines at the time of the study, pregnant women with unknown HIV status should have been offered provider-initiated rapid HIV testing and counselling. [7] Women who tested HIV-positive should have been given nevirapine (an intra-partum dose for themselves and a post-natal course for their infant). Lifelong ART, provided free, was recommended for pregnant women with a CD4 count ≤350 cells/mm^3^ and could be initiated within two days of diagnosis. [7], [8].

Naivasha Hospital policy at the time stated that, immediately following diagnosis with HIV in antenatal care (ANC), women should have been accompanied by the ANC nurse to the on-site HIV clinic for clinical staging, a two-week prescription of antenatal zidovudine (AZT) and cotrimoxazole, and referral to the on-site laboratory for CD4 count testing and then on to the pharmacy to collect their medications. Upon registration clients were asked to return within one week for a CD4 count test and then again two weeks later for the test result.

### Enrolment and Follow-up

#### Cohort study

Between January and September 2010, a consecutive sample of all women diagnosed with HIV who reported having been previously unaware of their HIV status and were ≥15 years attending Naivasha Hospital pregnancy-related services were invited to join the study cohort. Enrolment into the cohort took place on the same day that women were found to be eligible i.e. either on the day of their HIV diagnosis or during a repeat visit to pregnancy-related services.

Face-to-face questionnaires were administered to women at enrolment covering the woman’s pregnancy history, experience of HIV illness, testing and care, knowledge and attitudes about HIV care and treatment, and social support. For participants recruited within ANC, research staff were asked to escort study participants to the HIV clinic immediately following their study enrolment.

A follow-up questionnaire was administered by telephone or in person at least three months following enrolment to elicit information from women on pregnancy-related and HIV services accessed since diagnosis, disclosure and levels of social support, and recent illness.

In addition, researchers searched the registration books for attendance by participants at Naivasha Hospital and five additional HIV clinics where women were most often referred for care.

Attrition at each step along the pathway to HIV care and treatment was assessed using health facility records. Univariable logistic regression analysis was used to assess which factors were associated with registration at an HIV clinic within 90 days of HIV diagnosis. All variables associated with the outcome (p<0.10) were included in a multivariable logistic regression model, except for ‘personally knowing someone with HIV’, which was excluded due to colinearity with ‘personally knowing someone who has died from AIDS’.

Quantitative data were double-entered into Epi-Data Version 3.1 and any errors resolved. Analysis used Stata 10.1 (Stata Corp, College Station, Texas).

#### Qualitative sub-study

Face-to-face semi-structured qualitative interviews were carried out to explore the factors affecting women’s access to HIV-related services. Attempts were made to contact 38 cohort participants for an interview, of whom half (n = 19) were interviewed. Initially participants with no/low attendance at the HIV clinic were selected but due to difficulties contacting and arranging face-to-face interviews with women with low attendance, the sampling strategy was broadened to also include women with higher HIV clinic attendance.

Interviews lasted 40–90 minutes. Domains of inquiry included: living situation, experience of HIV diagnosis, social support, experiences of discrimination, and health-seeking behaviours. Reasons given by participants for not attending services were probed in detail.

All interviews were carried out by local interviewers in Swahili and digitally recorded, transcribed and then translated into English for analysis. Transcripts were read repeatedly specifically to identify emerging taxonomies and themes. A coding scheme was derived based initially on findings from a literature review and exploratory focus group discussions, and refined based on themes emerging in the interviews. Following the initial analysis, matrices were created in Excel to allow cross-group comparisons between women displaying different attendance patterns at HIV-related services. Continuous and iterative hypothesis generation and testing throughout the analysis allowed for continual refinement of themes and ensured that the insights provided by the data could be captured.

Qualitative data were managed using NVivo Version 8 (QSR International Pty Ltd. 2008).

## Results

### Cohort Study Enrolment

**Figure 1:**
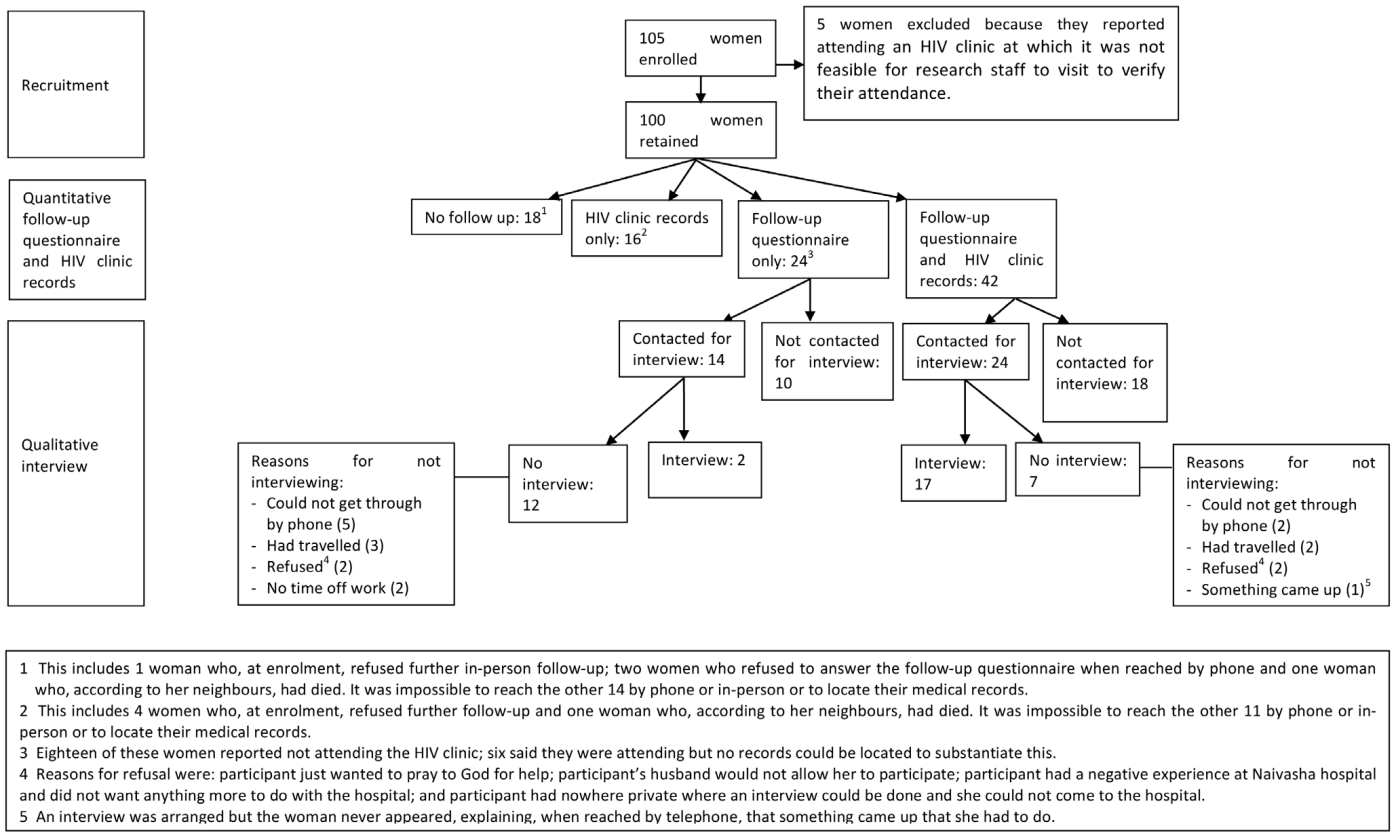
Cohort recruitment and follow-up.

Figure 1 shows the flow of participants in the cohort.

During the study timeframe, 110 women in Naivasha Hospital were eligible for study participation of whom 105 (95%) were invited to participate. The other five women received their HIV diagnosis on days when no research staff were at the hospital. All women invited to participate in the study consented to take part. Five women were excluded at the time of follow-up because quantitative analyses on HIV clinic attendance were based on health facility records and they reported having attended an HIV clinic that was too far away for the study team to access their records.

Of the 100 women in the cohort, 66 (66%) completed a structured follow-up questionnaire: 18 in person and 48 by telephone. Median time between enrolment and follow-up was 96.5 days (inter-quartile range (IQR): 93–110 days). No follow-up information was available for 18 (18%) women and a further 16 (16%) could not be reached by telephone or in person even though they had registered at an HIV clinic and their HIV clinic records were accessed. There were no significant differences in the baseline characteristics of women completing the follow-up questionnaire versus those who could not be reached, including HIV-related symptoms, distance from home to the hospital, and prior contact with people living with HIV (see Table S1).

### Cohort Participant Characteristics

Among 100 women enrolled in the cohort, the median age at enrolment was 26 years (IQR: 23–30) and the majority of women were married (Table 1). Most women had no more than primary education and just over half were in paid employment. The median gestational age at time of HIV diagnosis was 28 weeks. Women reported a median travel time of 35 minutes (IQR: 30, 60) to reach the hospital from their home, and 58% reported having to pay for transport.

**Table 1:** Factors Associated With Attendance At The HIV Clinic Within 90 Days Of HIV Diagnosis Among Women Diagnosed With HIV In Pregnancy-Related Services.

Description	N	Attended HIV clinic (row %)	UnAdjOR*	95%CI	AdjOR*	95%CI
*Age*			P = 0.980			
15–19	8	4 (50.0)	0.88	0.18, 4.17		
20–24	30	16 (53.3)	1.00			
25–29	35	20 (57.1)	1.17	0.44, 3.11		
30–44	27	15 (55.6)	1.09	0.38, 3.11		
*Education*			P = 0.521			
None	2	1 (50.0)	0.72	0.04, 12.08		
Some primary	62	36 (58.1)	1.00			
Some secondary	29	16 (55.2)	0.89	0.37, 2.16		
Post-secondary	7	2(28.6)	0.29	0.05, 1.61		
*Marital status*			P = 0.283			
Not married (single/widowed/separated)	28	13 (46.4)	1.00			
Married	72	42 (58.3)	1.62	0.67, 3.89		
*Employment*			P = 0.746			
Unemployed	44	25 (56.8)	1.00			
Employed	56	30 (53.6)	0.88	0.40, 1.94		
*Gravidity*			P = 0.554			
One	29	15 (51.7)	1.00			
Two	22	12 (54.5)	1.12	0.37, 3.40		
Three	31	20 (64.5)	1.70	0.60, 4.78		
Four+	18	8 (44.4)	0.75	0.23, 2.43		
*Timing of 1^st^ ANC*			P = 0.775			
8–21 weeks	13	7 (53.8)	1.00			
22–27 weeks	8	6 (75.0)	2.57	0.37, 17.83		
28–34 weeks	31	20 (64.5)	1.56	0.42, 5.81		
35–39 weeks	7	4 (57.1)	1.14	0.18, 7.28		
*Timing of HIV diagnosis*			P = 0.688			
≤28 weeks gestation (ANC)	63	34 (54.0)	1.00			
29+ weeks gestation (ANC)	30	18 (60.0)	1.28	0.53, 3.09		
Delivery	7	3 (42.3)	0.64	0.13, 3.10		
*Location of HIV diagnosis*			P = 0.788			
ANC	91	50 (54.9)	1.00			
Delivery	8	4 (50.0)	0.82	0.29, 3.48		
*Travel time from home to clinic*			P = 0.583			
60+ minutes	24	12 (50.0)	1.00			
<60 minutes	76	43 (56.6)	1.25	0.56, 2.78		
*Cost of travel to HIV clinic*			P = 0.015		P = 0.032	
Not having to pay a transport fare^1^	58	26 (44.8)	1.00		1.00	
Having to pay a fare	42	29 (69.0)	2.75	1.19, 6.32	2.73	1.09, 6.84
*HIV symptoms* 2			P = 0.039		P = 0.202	
None	60	28 (46.7)	1.00		1.00	
At least one	40	27 (67.5)	2.37	1.03, 5.46	1.83	0.72, 4.62
*Ever seen anyone with HIV*			P = 0.125			
No	16	6 (37.5)	1.00			
Yes	84	49 (58.3)	2.33	0.78, 7.02		
*Personally know anyone with HIV*			P = 0.089			
No	31	13 (41.9)	1.00			
Yes	68	41 (60.3)	2.10	0.89, 4.98		
*Personally know anyone who died of AIDS*			P = 0.037		P = 0.216	
No	16	5 (31.3)	1.00		1.00	
Yes	84	50 (59.5)	3.24	1.03, 10.15	2.21	0.63, 7.80
*Ever cared for anyone with HIV*			P = 0.008		P = 0.021	
No	72	34 (47.2)	1.00		1.00	
Yes	26	20 (76.9)	3.73	1.34, 10.36	3.67	1.22, 11.09
*Enough information to decide whether or not to test* 3			P = 0.064		P = 0.087	
No	13	4 (30.8)	1.00		1.00	
Yes	86	50 (58.1)	3.13	0.89, 10.94	3.61	0.83, 15.71
*Self-perceived ability to refuse to test*			P = 0.710			
No	62	35 (56.5)	1.00			
Yes	38	20 (52.6)	0.86	0.38, 1.93		
*Receipt of PMTCT prophylaxis*			P = 0.245			
Maternal and infant	45	23 (51.1)	1.00			
Maternal only	15	6 (40.0)	0.64	0.19, 2.09		
Infant only	31	19 (61.3)	1.51	0.60, 3.84		
No prophylaxis	9	7 (77.8)	3.35	0.63, 17.90		

*P-values relate to heterogeneity from a likelihood ratio test.

1 Participants who did not have to pay for transport walked to the hospital.

2 Participants were asked if they had experienced any of the following symptoms within the previous six months: diarrhoea; big problems with memory or concentration that interfered with normal activities; cough for more than two weeks; high fever; swollen glands; a yeast infection in the mouth or vagina (thrush); numbness, tingling or burning sensations in the arms, legs, hands or feet; substantial weight loss; or a skin rash.

3 Within a series of questions regarding pre-test counselling, women were asked “Did you feel that you were given enough information to decide whether or not to have an HIV test?”.

### Qualitative Sub-study Participant Characteristics

The 19 women who participated in the qualitative sub-study were more likely than the 81 who did not to have started attending ANC before 28 weeks gestation (75% vs. 26%; p = 0.001), more likely to have registered at the HIV clinic within 90 days of their HIV diagnosis (74% vs. 48%; p = 0.045), and more likely to have returned to the HIV clinic for a second visit (58% vs. 33%; p = 0.047).

### Client Attrition between Testing HIV-positive and Attending HIV-related Services

**Figure 2:**
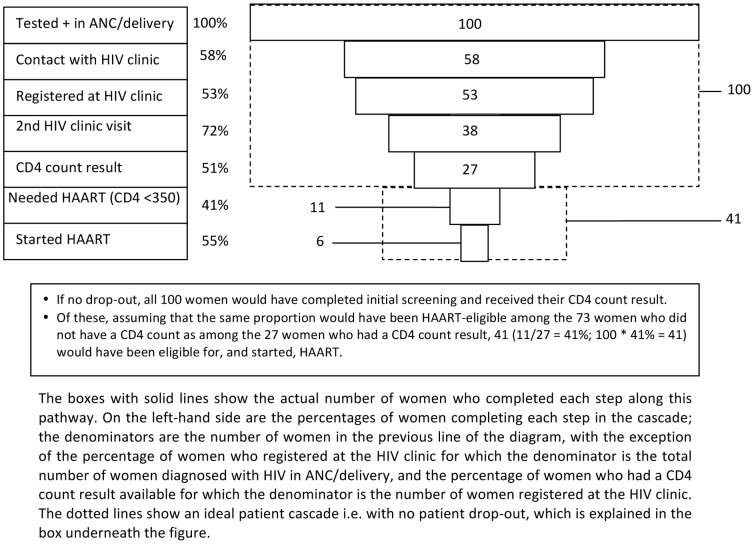
Client attrition along the pathway to long-term HIV care and treatment services.

Figure 2, based on health facility records, shows client attrition along the pathway to HIV care and treatment services within 90 days of diagnosis with HIV. If no health facility record could be found, it was assumed that the participant had not enrolled in HIV care and treatment.

Client attrition was substantial at each step of this pathway: 47/100 (47%) women were lost between diagnosis and registration at an HIV clinic; a further 26/53 (49%) of those registering at the HIV clinic did not have a CD4 count result in their file. Only 27 of the original 100 women (27%) had a CD4 count result. Among 11 ART-eligible women, a further 5 (45%) were lost between receiving their CD4 count and actually starting ART. Overall, only six women started ART (15% of the 41 women estimated as likely to have been ART-eligible).

### Delays Along the Pathway to HIV Care and Treatment

Among the 53 women who had registered at an HIV clinic during the 90-day follow-up period, 32 (60%) registered on the day of their HIV diagnosis of whom 12 (38%) did not return to the HIV clinic again during the follow-up period. The median time to registration at the HIV clinic among the remaining women who registered at the HIV clinic within 90 days was 21 days (IQR: 11–63).

The median time between registration and having blood taken for a CD4 count was six days (IQR: 0–17). For ART-eligible women, the median time between this blood-draw and initiating ART was 28 days (IQR: 0–37), and the median number of appointments between the visit at which they received their CD4 count result and being started on ART was two (Range: 0–5). Among ART-eligible women who successfully started ART within the 90-day follow-up period, the median time between diagnosis and initiating ART was 48.5 days (IQR: 32–76).

### Factors Associated with Uptake of HIV-related Services

Table 1 shows baseline factors associated with HIV clinic registration within 90 days of diagnosis among the 100 women in the cohort. As with Figure 2, this is based on health facility records. In the univariable analysis these factors were: having cared for someone with HIV (OR: 3.73; 95%CI: 1.34, 10.36; p = 0.008), having to pay for transport to the hospital (OR: 2.75; 95%CI: 1.19, 6.32; p = 0.015), personally knowing someone who died of AIDS (OR: 3.24; 95%CI: 1.03, 10.15; p = 0.037), and having experienced at least one HIV-related symptom in the six months prior to enrolment (OR: 2.37; 95%CI: 1.03, 5.46; p = 0.039). There was also weak evidence of an association with reporting having received enough information to decide to have an HIV test (OR: 3.13; 95%CI: 0.89, 10.94; p = 0.064) and with personally knowing someone with HIV (OR: 2.10; 95%CI: 0.89, 4.98; p = 0.089).

In the multivariable model, factors remaining independently associated with the outcome were: having cared for someone with HIV (aOR:3.67; 95%CI: 1.22, 11.09; p = 0.021) and having to pay for transport to the hospital (aOR:2.73; 95%CI: 1.09, 6.32; p = 0.032). There was also weak evidence of an independent association between the outcome and having received enough information to decide to have an HIV test (aOR:3.61; 95%CI: 0.83, 15.71; p = 0.087).

In the follow-up questionnaire administered to 66 women, 18 (27%) reported not having registered at an HIV clinic. The three most commonly cited reasons for not having registered at an HIV clinic were: women not knowing that they had to register (4/18; 22%), not having had time to register (3/18; 17%), and not feeling ready to do so (2/18; 11%).

Of the 53 women who delivered during the study period and had registered at the HIV clinic, 15 (28%) stopped attending the HIV clinic for at least eight weeks around the time of delivery.

### Qualitative Exploration of Reasons Underlying Women’s Decisions Regarding Accessing HIV-related Services

The semi-structured interviews corroborated many of the quantitative findings presented above. Three main areas were reported to have affected women’s uptake of HIV services for their own care: social support, interactions with health workers and health services-related factors.

#### Social support

Supportive partners, family and friends facilitated uptake of care by helping around the house, reminding women to take their drugs and providing emotional support:

“*[My husband] tells me that I shouldn’t stress myself…. [He says] ‘Stop stressing yourself… there are people who love you’.*” (Participant #126, Age 31).

Conversely, many women reported that following disclosure to their partner they had never again discussed their HIV status with him or any other friend or family member, primarily because they perceived a lack of willingness to talk about their HIV status with them. As one woman explained:

*“I went to test. [After the test]… I made a phone call to my husband. He told me that it is my problem. He said he doesn’t have [HIV]… I told him and he ran away, and when he came back he told me not to tell him that information again.”* (Participant #218, Age 21).

Some women reported that their husbands did not want them to disclose their status to anyone else. This lack of communication and low level of disclosure beyond partners left some women feeling isolated as they struggled to come to terms with their HIV diagnosis.

Women’s perceptions of HIV-related attitudes suggested that, for most, the potential negative social consequences of disclosure still outweighed perceived benefits, which contributed to low levels of attendance at HIV clinics and support groups.

#### Interactions with health workers

Health workers might be considered another potentially important source of support for women with HIV. Yet many women described suboptimal communications with health workers. In the context of HIV counselling, many women reported feeling unprepared for being tested:

*“I felt like she just ambushed me and tested me… people are supposed to be given counselling… so that if [the test result] is positive… you won’t have to worry a lot.”* (Participant #228, Age 32).

In addition, many women reported being “*talked to badly*” by reception staff at the HIV clinic, with some feeling “*a little despised*”. They found this particularly intimidating during early visits to the HIV clinic and some women reported being afraid to attend the clinic again if they had missed an appointment for fear of being reprimanded.

Other instances where interactions with health workers negatively affected uptake of services were also reported. One woman travelled to her rural home for three months where the HIV clinic refused to register her as she lived in Naivasha so she only began attending services when she returned to Naivasha.

*“They said I couldn’t [attend the local HIV clinic]. I must be at a permanent place because of my status… I was not from there so they said they wouldn’t do a CD4 for me.”* (Participant 116, Age 33).

Another participant was told not to start ART until she had completed three months of treatment for tuberculosis, which she duly did, even though this delay was contrary to national guidelines for HIV treatment initiation.

In several cases, a sudden change in HIV clinic attendance patterns – from regular attendance at scheduled appointments to non-attendance – came immediately following a negative experience within the hospital. One woman, for example, dropped out of care when she was asked to repeat (and re-pay for) her CD4 count test as her result had been lost. Another woman stopped attending following a miscarriage. In addition to associating her miscarriage with having started ART a week before it occurred, she described staff refusal to attend to her during the stillbirth. At the time of the interview, she had not been to the HIV clinic for five months, meaning that she was not receiving cotrimoxazole as recommended.

Conversely, one woman began attending HIV services months after her HIV diagnosis based on a positive interaction with a health worker: she had attempted to abandon her baby and commit suicide but she was caught, arrested, and taken to the hospital where she reported having received great support and encouragement through counselling from the hospital matron.

#### Health services-related factors

Reflecting the quantitative findings, costs, including transport costs, were frequently mentioned as a barrier to hospital attendance.

*“I lacked money to start on ARVs because I was told that I had to have 250 shillings ($2.87) to begin the clinic. It took me time because everyone had deserted me… my neighbours, husband… I had no friends and no money. So it forced me to sell my baby’s blanket so that I could afford to start the clinic.”* (Participant #215, Age 22).

Many women reported that they had delayed or not had a CD4 count blood-draw due to its cost (usually approximately KShs170 ($1.95)). Conversely, antiretrovirals being free proved a very strong motivational factor for many women to attend HIV services.

Women described difficulties navigating the pathway between HIV testing in pregnancy-related services and long-term HIV care and treatment. Women described having to visit multiple departments in a single day with long waiting times and having to visit the hospital on many different days:

*“They make you wait the whole day and make you come here another time for something else, it is not good.”* (Participant #115, Age 20).

Additional challenges included difficulty in understanding directions, poor understanding of the patient pathway and restricted clinic hours.

## Discussion

A lower proportion (53%) of women diagnosed with HIV in pregnancy in this cohort registered at the HIV clinic than in previously published studies (62–85%) from elsewhere in sub-Saharan Africa, [9]–[12] although this might be partially explained by this study’s relatively short follow-up period. The higher proportion of women registering at an HIV clinic in this study compared to the retrospective study we carried out in Naivasha Hospital can be largely attributed to improvements in attendance over time and the ability of the prospective cohort to capture women’s attendance at a range of HIV clinics outside Naivasha Hospital.

The uptake of ART among known-eligible women diagnosed with HIV in pregnancy-related services reported in previous studies in sub-Saharan Africa has varied enormously (12–95%). [9], [10], [12]–[19] A recent meta-analysis that included both observational and intervention studies estimated that 68% of women diagnosed with HIV in pregnancy had a CD4 cell count done and that 61% of the women who were eligible for ART started treatment. [20] Due to attrition at each step along the pathway to treatment, our study found low uptake of ART in comparison to many other studies. [4] The scale of and reasons underlying attrition from long-term HIV care and treatment services around the time of delivery warrant further study in larger studies.

The qualitative finding that lack of social support impeded uptake of long-term HIV care and treatment services echoes previous studies that have found higher levels of social support to facilitate uptake of PMTCT services and adherence to ART. [21], [22] Although nurse escorts to the HIV clinic at Naivasha Hospital might have increased registration at this clinic immediately following diagnosis with HIV in pregnancy-related services, the high proportion of women who never returned for a subsequent visit to the HIV clinic suggests the need for on-going support for women to ensure uptake of CD4 count testing as well as on-going care and treatment services. The association between having cared for someone living with HIV and successful linkage to the HIV clinic is a new finding. Previous studies have shown close personal contact with someone with HIV to be associated with changes in HIV-related risk behaviours, [23], [24] but this has not previously been explored with regard to uptake of HIV care and treatment services.

Although an insufficient measure for informed choice, the weak association between women reporting having received enough information to decide whether or not to take an HIV test and registering at an HIV clinic suggests that current counselling in some settings may be insufficient for preparing pregnant women for a potential HIV diagnosis, with implications for their subsequent uptake of care. A study in Uganda found that some women perceive provider-initiated HIV testing and counselling within antenatal care as compulsory and they do not fully understand its potential consequences, [25] but this remains an underexplored area.

Finally, this study confirms previous studies’ findings that complicated patient pathways, [11], [26], [27] low knowledge among clients of the need to attend the HIV clinic, [27] transportation costs, [28]–[33] and negative experiences in health facilities may adversely affect future uptake of services. [28], [34], [35].

### Limitations

Limitations of this study include its relatively small sample size. We may have over-estimated client losses if some of the 18 women with no follow-up information accessed HIV care outside the study area. On the other hand, observation showed that the hospital’s policy that a nurse from the ANC should accompany any HIV-positive woman to the HIV clinic was not always followed so researchers escorting women to the HIV clinic will have improved their initial linkage into care.

The difficulty arranging qualitative interviews with women who had poorer hospital attendance meant that interview participants under-represented this group. Women’s primary reason for refusing to be interviewed was that they had travelled to rural areas and would not return for weeks or months, which would equally have impeded their hospital attendance.

### Conclusions and Recommendations

Only 15% of women estimated to be ART-eligible in this cohort started ART within 90 days of their HIV diagnosis. Recommendations for increasing uptake of long-term HIV care and treatment services include: appropriate counselling that allows sufficient time and information for women to make informed decisions about if and when to use services; simplifying the pathway to care by reducing the required number of visits and increasing accessibility of CD4 testing, ideally using point-of-care CD4 testing; ensuring the daily availability of ART services within ANC (this is being promoted by the Kenyan government and the WHO); [8], [36] ensuring adequate staffing within ANC, delivery and the HIV clinic; linking women to the HIV clinic post-delivery; and improving the quality of interactions between health workers and clients.

The benefits to an individual of timely access to care, coupled with the increased costs to the health system incurred through delayed access, draw attention to the need to address the factors identified as impediments to prompt linkage into HIV care and treatment services.

## Supporting Information

Table S1 **Participant characteristics by follow-up status.**
(DOCX)
