# Supplementary material for: Linking Women Who Test HIV-Positive in Pregnancy-Related Services to HIV Care and Treatment Services in Kenya: A Mixed Methods Prospective Cohort Study
Source: PLoS One. 2014 Mar 19;9(3):e89764. doi: 10.1371/journal.pone.0089764 (PMC3960101; doi:10.1371/journal.pone.0089764)
Supplement: Table S1 — Participant characteristics by follow-up status. (DOCX) [file pone.0089764.s001.docx]

Table S1: Participant characteristics by follow-up status

| **Description** | **Total** | **Completed follow-up questionnaire (n=66)**  **(col %)** | | **Did not complete follow-up questionnaire (n-34)**  **(col %)** |
| --- | --- | --- | --- | --- |
| *Age* |  |  | P=0.513 | |
| 15-19 | 8 | 7 (11%) | 1 (3%) | |
| 20-24 | 30 | 18 (27%) | 12 (35%) | |
| 25-29 | 35 | 22 (33%) | 13 (38%) | |
| 30-44 | 27 | 19 (29%) | 8 (24%) | |
| *Education* |  |  | P=0.599 | |
| None | 2 | 2 (3%) | 0 (0%) | |
| Some primary | 62 | 39 (59%) | 23 (68%) | |
| Some secondary | 29 | 19 (29%) | 10 (29%) | |
| Post-secondary | 7 | 6 (9%) | 1 (3%) | |
| *Marital status* |  |  | P=0.491 | |
| Widowed | 3 |  |  | |
| Single | 17 | 17 (26%) | 11 (32%) | |
| Separated | 8 |  |  | |
| Married | 72 | 49 (74%) | 23 (68%) | |
| *Employment* |  |  | P=0.832 | |
| Unemployed | 44 | 30 (45%) | 14 (41%) | |
| Employed | 56 | 36 (55%) | 20 (59%) | |
| *Gravidity* |  |  | P=0.816 | |
| One | 29 | 21 (32%) | 8 (24%) | |
| Two | 22 | 14 (21%) | 8 (24%) | |
| Three | 31 | 19 (29%) | 12 (35%) | |
| Four + | 18 | 12 (18%) | 6 (18%) | |
| *Timing of 1^st^ ANC* |  |  | P=0.174 | |
| 8-21 weeks | 13 | 11 (27%) | 2 (11%) | |
| 22-27 weeks | 8 | 7 (17%) | 1 (6%) | |
| 28-34 weeks | 31 | 20 (49%) | 11 (61%) | |
| 35-39 weeks | 7 | 3 (7%) | 4 (22%) | |
| *Timing of HIV diagnosis* |  |  | P=0.228 | |
| ≤ 28 weeks gestation (ANC) | 63 | 45 (68%) | 18 (53%) | |
| 29+ weeks gestation (ANC) | 30 | 16 (24%) | 14 (41%) | |
| Delivery | 7 | 5 (8%) | 2 (6%) | |
| *Travel time from home to clinic* |  |  | P=1.000 | |
| 60+ minutes | 57 | 38 (58%) | 19 (56%) | |
| < 60 minutes | 43 | 28 (42%) | 15 (44%) | |
| *Location of HIV diagnosis* |  |  | P=0.711 | |
| ANC | 91 | 59 (91%) | 32 (94%) | |
| Delivery | 8 | 6 (9%) | 2 (6%) | |
| *Cost of travel to HIV clinic* |  |  | P=0.201 | |
| Not having to pay a transport fare^1^ | 58 | 35 (53%) | 23 (68%) | |
| Having to pay a fare | 42 | 31 (47%) | 11 (32%) | |
| *HIV symptoms^2^* |  |  | P=0.289 | |
| None | 60 | 37 (56%) | 23 (68%) | |
| At least one | 40 | 29 (%44) | 11 (32%) | |
| *Ever seen anyone with HIV* |  |  | P=1.000 | |
| No | 16 | 11 (17%) | 5 (15%) | |
| Yes | 84 | 55 (83%) | 29 (85%) | |
| *Personally know anyone with HIV* |  |  | P=0.360 | |
| No | 31 | 23 (35%) | 8 (24%) | |
| Yes | 68 | 43 (65%) | 25 (76%) | |
| *Personally know anyone who died of AIDS* |  |  | P=0.159 | |
| No | 16 | 8 (12%) | 8 (24%) | |
| Yes | 84 | 58 (88%) | 26 (76%) | |
| *Ever cared for anyone with HIV* |  |  | P=1.000 | |
| No | 72 | 48 (73%) | 24 (75%) | |
| Yes | 26 | 18 (27%) | 8 (25%) | |
| *Enough information to decide whether or not to test*^3^ |  |  | P=0.760 | |
| No | 13 | 8 (12%) | 5 (15%) | |
| Yes | 86 | 57 (88%) | 29 (85%) | |
| *Self-perceived ability to refuse to test* |  |  | P=0.668 | |
| No | 62 | 42 (64%) | 20 (59%) | |
| Yes | 38 | 24 (36%) | 14 (41%) | |
| *Receipt of PMTCT prophylaxis* |  |  | P=0.690 | |
| Maternal and infant | 45 | 27 (41%) | 18 (53%) | |
| Maternal only | 15 | 11 (17%) | 4 (12%) | |
| Infant only | 31 | 21 (32%) | 10 (29%) | |
| No prophylaxis | 9 | 7 (11%) | 2 (6%) | |

p-values are based on a Fisher’s exact test.
